# Synthesis of new-type, cost-effective and insensitive energetic materials *via* nitration of solid bituminous hydrocarbons†

**DOI:** 10.1039/d4ra06329e

**Published:** 2024-11-11

**Authors:** Sahar Abdolahi, Mohammad Soleiman-Beigi

**Affiliations:** a Department of Chemistry, Faculty of Basic Sciences, Ilam University P.O. Box 69315516 Ilam Iran SoleimanBeigi@yahoo.com m.soleimanbeigi@ilam.ac.ir

## Abstract

A global trend for the development of energetic materials using various sources is promoted by researchers annually. Solid bituminous hydrocarbons can play a key role in carbon science as abundant, low-cost, and mineral carbonaceous substrates. This study focuses on the design and synthesis of a series of new energetic materials from natural asphalt (NA), petroleum pitch (PP) and petroleum bitumen (PB) as industrial and available solid bituminous hydrocarbons. Energetic materials NA-NO_2_, PP-NO_2_ and PB-NO_2_ were synthesized through the nitrification reaction. The heat of combustion, thermal behaviors and FTIR, elemental, BET, UV-vis, SEM, EDX-map, AFM, GC-MS and TG-DSC analyses were applied to identify and confirm that all were prepared successfully. Further, the physicochemical and energy properties of NA-NO_2_, PP-NO_2_ and PB-NO_2_ were calculated using EMDB V 1.0 software. Thermal analysis showed thermal stability and insensitivity of NA-NO_2_, PP-NO_2_ and PB-NO_2_ toward mechanical stimuli. The combustion heats of NA-NO_2_, PP-NO_2_ and PB-NO_2_ were measured using a calorimeter bomb *via* the ASTM D240 method and evolved high amounts of energy of 23 500, 23 450 and 23 360 kJ kg^−1^, respectively. The density of NA-NO_2_ was measured using the ASTM-D8176 test and confirmed to be 0.5 g cm^−3^, which can be considered the lightest energetic material. Based on the conducted studies and analyses, new energetic materials synthesized based on solid bituminous hydrocarbons are classified as first-generation energetic materials.

## Abstract 2

A global trend for the development of energetic materials using various sources is promoted by researchers annually.

## Graphical abstract

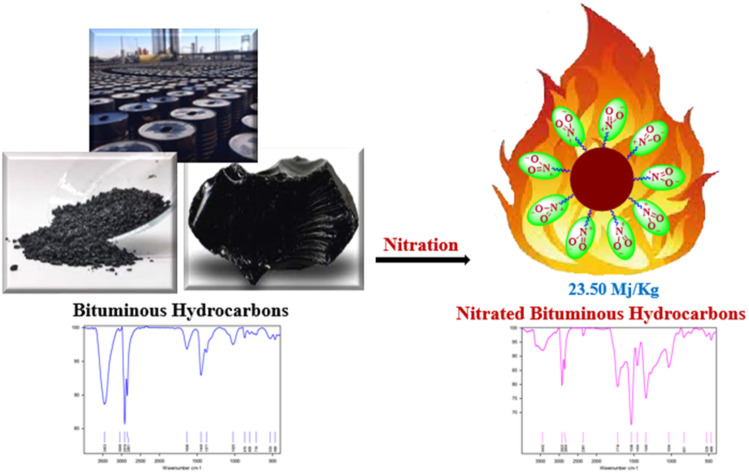

## Introduction

Nowadays, organic nitro compounds are widely used in industrial applications as multilateral synthetic intermediates.^1^ These compounds are applied as key substrates for the preparation of useful materials such as building blocks to construct complex molecules in biological industries, agrochemicals, explosives, dyes, pharmaceuticals, polymers, perfumes, synthetic blocks, plastics, and manures and as catalytic substrates for organic reactions.^2–4^ In the past few decades, the study of new energetic materials has attracted the attention of many research groups due to their importance and value in propellants, explosives, and pyrotechnics.^5–9^ These materials can instantly release tremendous energy in response to a certain stimulus.^10–12^ The nitro group is the most important moiety of energetic organic materials, which is generally created *via* nitrolysis, direct nitration, oxidation, and rearrangement reactions that include nitrite anion or nitronium cation.^13^ Nitroaromatic compounds are a group of organic compounds with energetic groups that can decompose, ignite and explode due to heat and impact. Therefore, before the synthesis of these materials, it is necessary to pay attention to their thermochemical properties for storage, safe handling, and transportation.^14^ Energetic materials are categorized into three generations. The first-generation compounds such as 2,4,6-trinitrotoluene (TNT) are composed of benzene skeletons, which suffer from poor detonation properties. The second-generation energetic materials include compounds such as 1,3,5,7-tetranitrotetraazacyclooctane (HMX) and 1,3,5-trinitrotriazocyclohexane (RDX), which are derived from cyclic aliphatic and nitramino structures, and they have poor stability. The third-generation compounds such as 2,4,6,8,10,12-hexanitro-2,4,6,8,10,12-hexaazatetracyclododecane (CL-20) are derived from caged nitramino structures. These compounds have high sensitivity and favorable detonation properties.^15–18^

In recent years, some materials such as [60]fullerene polyglycidyl (C_60_-PG),^19,20^ bacterial cellulose (BC),^21^ carbon nanotubes (CNTs),^22^ cellulose-coated multiwalled carbon nanotube (C-coated MWCNT),^23^ cellulose,^24^ cellulose propionate,^25^ fullerene,^26^ polystyrene (PS),^27^ graphene oxide (GO)^28^ and single-walled carbon nanotubes (SCNTs)^29^ have been investigated as carbonaceous substrates for nitration reaction. They have been widely utilized due to their high specific surface area and the ability to graft groups on their surface area. However, most of these carbonaceous materials have limitations such as unavailability, high cost, poor stability, difficulty in nitration and low ratio of nitration.^24,25,27–31^ Additionally, a new method of thermal analysis was evaluated to determine the SIT self-ignition temperature of different bitumen nitrate mixtures. However, for nitration, bitumen mixed in NaNO_3_, Ni(NO_3_)_2_ and AgNO_3_ or AgI was prepared.^32^

Solid bituminous hydrocarbons such as natural asphalt (NA), petroleum pitch (PP), and petroleum bitumen (PB) are inexpensive, available and abundant materials that contain a high precursor of carbon (more than 70%). These carbon-containing materials are of interest for various applications such as dyes, road pavements, and building materials.^31,33–35^ NA is a heavy hydrocarbon and a type of bituminous material that is extracted in ready form through mineral extraction or from natural bitumen mines and resources. That contains about 80% carbon and 15% hydrogen, which is easily dissolved in tetrachloroethylene and carbon disulfide solvents.^31,36^ Also, recently NA support has been applied as a carbonaceous super adsorbent, which is used for rapid decolorization of aqueous dye solutions.^37^ Whereas, PP is one of the refinery petroleum derivatives and a complex mixture of polynuclear aliphatic and aromatic hydrocarbons, which has wide applications in the field of carbon fiber, feedstock for electrodes and supercapacitors.^33,34,38^ Moreover, PB is also one of the refinery petroleum derivatives, whose components include saturated and aromatic hydrocarbons, asphaltenes and resins.^27^ NA and PP are lumpy, black, and have a melting point above 180 °C.

Herein, in continuation of our previous research regarding the synthesis of new materials from natural asphalt, solid natural asphalt as a type of solid bituminous hydrocarbon has been nitrated for the synthesis of highly nitronatural asphalt (NA-NO_2_) under different conditions. In the following, we also nitrated petroleum pitch and petroleum bitumen for the synthesis of nitropetroleum pitch (PP-NO_2_) and nitropetroleum bitumen (PB-NO_2_) according to the natural asphalt nitrate conditions. Finally, the ability of these compounds in the field of energetic materials was evaluated and discussed.

## Results and discussion

### General procedure for nitration of solid bitumen hydrocarbons by HNO_3_/H_2_SO_4_

The synthesis method of NA-NO_2_, PP-NO_2_ and PB-NO_2_ is summarized as follows: first, 20 mL of H_2_SO_4_ was slowly added to a 100 mL round-bottom flask in an ice bath (∼−5 °C) containing 17.5 mL HNO_3_. Then, the mixture was stirred at room temperature for 15 min. In the next step, 2 g of the substrate was gradually added with stirring for 20 minutes. After 30 minutes of the reaction mixture, the temperature of the reaction was slowly increased to 60 °C and the reaction was allowed to continue for 5 hours. Subsequently, the reaction was completed, and 300 mL of distilled water was added to the reaction mixture. Then, the precipitate was filtered and washed several times with distilled water and finally dried at a temperature of 80 °C. In the end, 3.0, 2.75 and 2.60 g of brown powders of NA-NO_2_, PP-NO_2_ and PB-NO_2_ compounds were obtained, respectively.

### Nitro natural asphalt synthesis (NA-NO_2_)

**Scheme 1:**
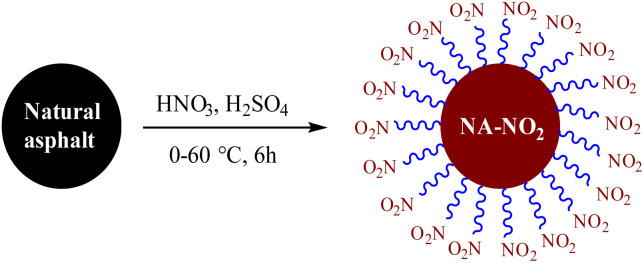
Nitration of natural asphalt.

To determine the optimal reaction conditions, various factors such as elemental analysis, color, weight gain, and flammability of product (NA-NO_2_) were assayed. First, solid natural asphalt powder, 150–200 mesh, was nitrated through nitration using different reagents. Finally, HNO_3_/H_2_SO_4_ as a common and available reagent was selected for nitration of natural asphalt in high yield (Table 1, entry 3). This reagent was preferred to others due to more nitrogen content, lower ash content, faster burning and higher yield of the obtained target product. The synthetic route of the NA-NO_2_ is shown in Scheme 1.

**Table 1:** Optimizing the reaction conditions for the synthesis of NA-NO_2_ in the presence of different reagents^a^

Entry	Substrate	Reagent(s) (ratio/mL)	CHNS analysis				Color	Flammability	Weight gain^b^ (g)
			% C	% N	% H	% S			
1	Natural asphalt	—	61.76	0.86	7.34	7.38	Black	—c	—
2	Natural asphalt	HNO_3_ (17.5)	70.85	5.61	3.44	5.43	Black	F	2.10
**3**	**Natural asphalt**	**HNO**_**3**_ **/H**_**2**_ **SO**_**4**_ **(17.5/20)**	**46.91**	**6.11**	**4.68**	**5.52**	**Light brown**	**F**	**3.0**
4	Natural asphalt	HNO_3_/H_2_SO_4_/AC_2_O (17.5/20/5)	49.76	6.80	4.52	1.42	Dark brown	F	2.20
5	Natural asphalt	HNO_3_/H_2_SO_4_/AC_2_O (17.5/20/10)	45.03	7.34	3.53	1.98	Brown	F	2.40
6	Natural asphalt	HNO_3_/H_2_SO_4_/P_2_O_5_ (17.5/20/10 g)	47.29	7.58	3.48	1.30	Light brown	F	2.42
7	Natural asphalt^d^	HNO_3_/H_2_SO_4_/CaCl_2_ (17.5/20/10 g)	—	—	—	—	Gray	—c	—
8	Natural asphalt^d^	NaNO_2_/KHSO_4_/H_2_O_2_ (1/0.8/0.03 g)	—	—	—	—	Black	—c	—
9	Petroleum pitch	—	82.27	1.03	6.70	4.88	Black	—c	—
10	Petroleum pitch	HNO_3_/H_2_SO_4_ (17.5/20)	48.99	8.18	2.53	6.33	Brown	F	2.75
11	Petroleum bitumen	HNO_3_/H_2_SO_4_ (17.5/20)	50.42	6.67	7.42	10.99	Brown	F	2.60

a Reaction Condition: Substrate (2 g), various reagents, 6 h, 0–60 °C.

b The weight of the obtained product after nitration 2 g of substrate.

c Not flammable.

d These methods were not nitrated.

**Fig. 1:**
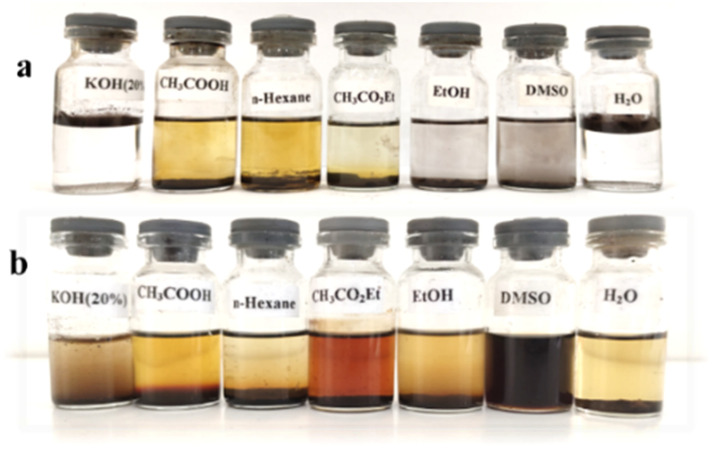
Solubility of natural asphalt (a) and NA-NO_2_ (b).

**Fig. 2:**
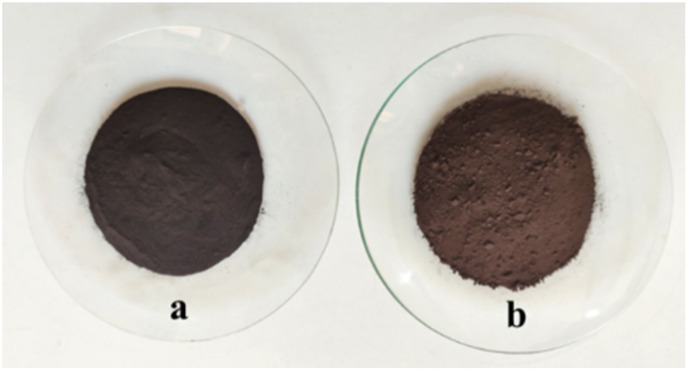
Color of natural asphalt (a) and NA-NO_2_ (b).

**Fig. 3:**
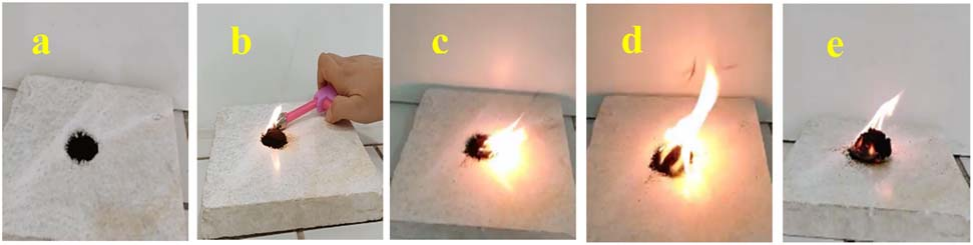
Continuous images of natural asphalt (a) burning using alighter (b–e).

Other reagents, including HNO_3_/H_2_SO_4_/CaCl_2_ and NaNO_2_/KHSO_4_/H_2_O_2_, were found to be ineffective in the nitrification of natural asphalt (Table 1, entries 7 and 8). Moreover, other nitrated reagents of entries 2 and 4–6 are mentioned in the ESI,†Table 1. The solubility and color of NA-NO_2_ were evaluated; it was found in light-brown color and completely soluble in aprotic solvents such as DMSO (Fig. 1 and 2). Additionally, NA-NO_2_ decomposed at temperatures above 300 °C. Although determining the exact active sites on natural asphalt for nitration is difficult, analyses and data show that both aromatic and aliphatic carbons in natural asphalt might be nitrated. Further, to gain insight into the burning rate of NA-NO_2_ high-speed images of the combustion process were recorded (Fig. 3). A lighter was used as the ignition source for the NA-NO_2_ flammability test.

In the continuation of research, petroleum pitch and petroleum bitumen as other available solid bituminous hydrocarbons were nitrated in parallel with natural asphalt powder in the same method. The results show that PP and PB were also successfully nitrated; based on elemental analysis, their nitrogen content was 8.18 and 6.67, respectively. They exhibited the same physical properties as NA-NO_2_. In addition, the burning test of PP-NO_2_ and PB-NO_2_ was investigated and demonstrated that they have poor flammability compared to NA-NO_2_ (Table 1, entries 10, 11).

After finding the best nitration reagent, the influence of natural asphalt amount, time and the ratio of acids (HNO_3_/H_2_SO_4_) on the efficiency of the reaction was assayed. Based on the nitrogen content and the weight of the obtained products, it can be concluded that the above factors affect the nitration reaction (Table 2).

**Table 2:** Optimization of the ratio of acids (HNO_3_/H_2_SO_4_) to natural asphalt for the synthesis of NA-NO_2_^a^

Entry	Natural asphalt (g)	Time (h)	HNO_3_/H_2_SO_4_ (mL)	CHNS				Color	Weight gain (g)
				% C	% N	% H	% S		
1	1	6	17.5/20	60.48	5.86	5.88	4.99	Brown	2.3
2	1	6	4/5	55.27	5.98	4.37	5.56	Black	2.17
3	1	6	2/3	56.55	5.37	4.38	5.36	Black	2.09
4	2	24	17.5/20	50.97	5.77	4.70	5.95	Brown	2.5
5	2	2	17.5/20	58.76	5.78	4.86	6.02	Black	2.4
**6**	**2**	**6**	**17.5/20**	**46.91**	**6.11**	**4.68**	**5.52**	**Light brown**	**3.0**

a Reaction Condition: natural asphalt (1–2 g), time (2–24 h), diverse ratio of acids, 0–60 °C.

### FTIR analysis

**Fig. 4:**
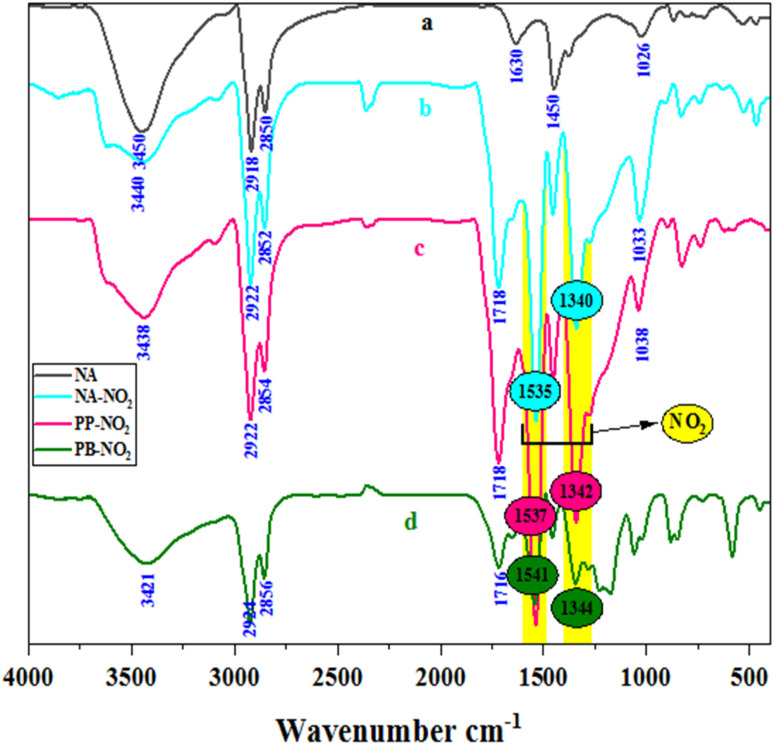
FTIR spectra of NA (a), NA-NO_2_ (b), PP-NO_2_ (c) and PB-NO_2_ (d).

FTIR spectra of NA (a), NA-NO_2_ (b), PP-NO_2_ (c) and PB-NO_2_ (d) are enumerated in Fig. 4. In spectra a-d, peaks around 1440–1600 cm^−1^ and 2850–2924 cm^−1^ are related to aromatic double bands and aliphatic C–H stretching vibrations, respectively. Besides, the stretching vibrations related to N–H and O–H groups were observed in 3421–3450 cm^−1^. When the nitration reaction occurs on solid bitumen hydrocarbons such as natural asphalt (a), the peaks at 1718 cm^−1^ (b and c) and 1716 cm^−1^ (d) corresponding to the CO group appear, which can be due to the oxidation of OH group and the hydrolysis of amide to acid. In the case of spectra (b–d), symmetric and asymmetric stretching vibrations of NO_2_ groups were revealed in 1340 and 1541 cm^−1^. NO_2_ groups confirmed that the nitration process was successfully carried out on solid bitumen hydrocarbons. Furthermore, the FTIR spectra of the obtained products *via* other nitration methods are provided in ESI, Fig. S1 and S2,† respectively.

### UV-vis analysis

**Fig. 5:**
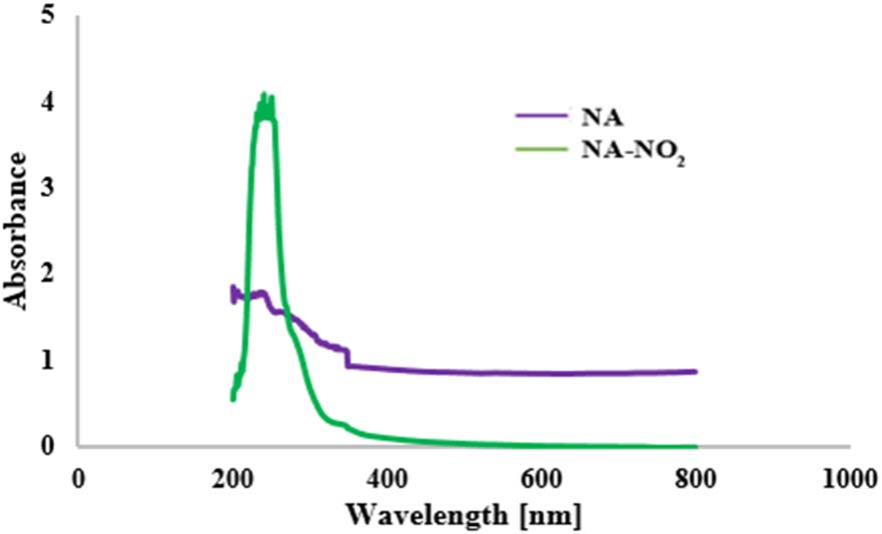
UV-visible absorption spectra for NA and NA-NO_2_.

The UV-vis spectra of NA and NA-NO_2_ are given in Fig. 5. The absorption behavior of NA-NO_2_, PP-NO_2_ and RB-NO_2_ compounds are quite similar and their absorption peak appears around 222–270 nm that it is mainly due to π–π* and n–π* transitions while an absorption peak was not observed for natural asphalt. This demonstrates that the absorption peak of the molecules can originate from nitrobenzene moiety. UV-vis spectra PP-NO_2_ and PB-NO_2_ are given in Fig. S3 and S4.†

### BET analysis

**Fig. 6:**
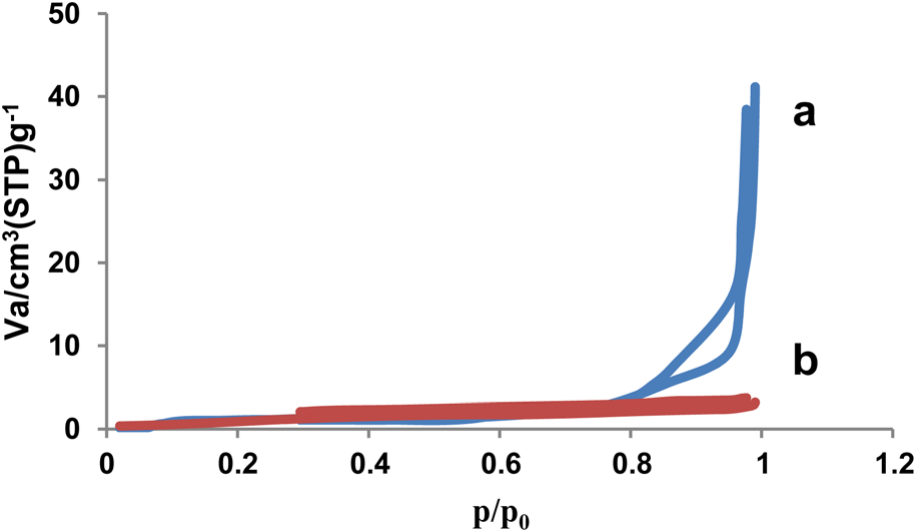
The N_2_ adsorption–desorption isotherm of NA (a) and NA-NO_2_ (b).

In order to determine the surface area, volume and pore diameter of the NA (a) and NA-NO_2_ (b), nitrogen adsorption–desorption analysis was used under standard temperature and pressure conditions. According to the IUPAC classification, the nitrogen adsorption–desorption diagram of NA-NO_2_ showed the type III isotherm (Fig. 6).

Based on BET data, surface area (*S*_BET_), total pore volume (*V*_total_) and pore diameter (*D*_BJH_) of NA were obtained as 10.49 m^2^ g^−1^, 0.132 cm^3^ g^−1^ and 10.65 nm, respectively. The surface area of natural asphalt decreased to 4.34 m^2^ g^−1^ after nitration. The total pore volume and average pore diameter of the NA-NO_2_ were evaluated as 0.0065 cm^3^ g^−1^ and 1.66 nm, respectively. These results showed that NA-NO_2_ was successfully synthesized and confirmed the presence of NO_2_ groups on the natural asphalt molecular structure (Table 3).

**Table 3:** Textural properties of NA and NA-NO_2_ obtained by nitrogen adsorption–desorption analysis

Sample name	*S*_BET_ (m^2^ g^−1^)	*V*_Total_ (cm^3^ g^−1^)	*D*_BJH_ (nm)
NA	10.49	0.132	10.65
NA-NO_2_	4.34	0.0065	1.66

### SEM analysis

**Fig. 7:**
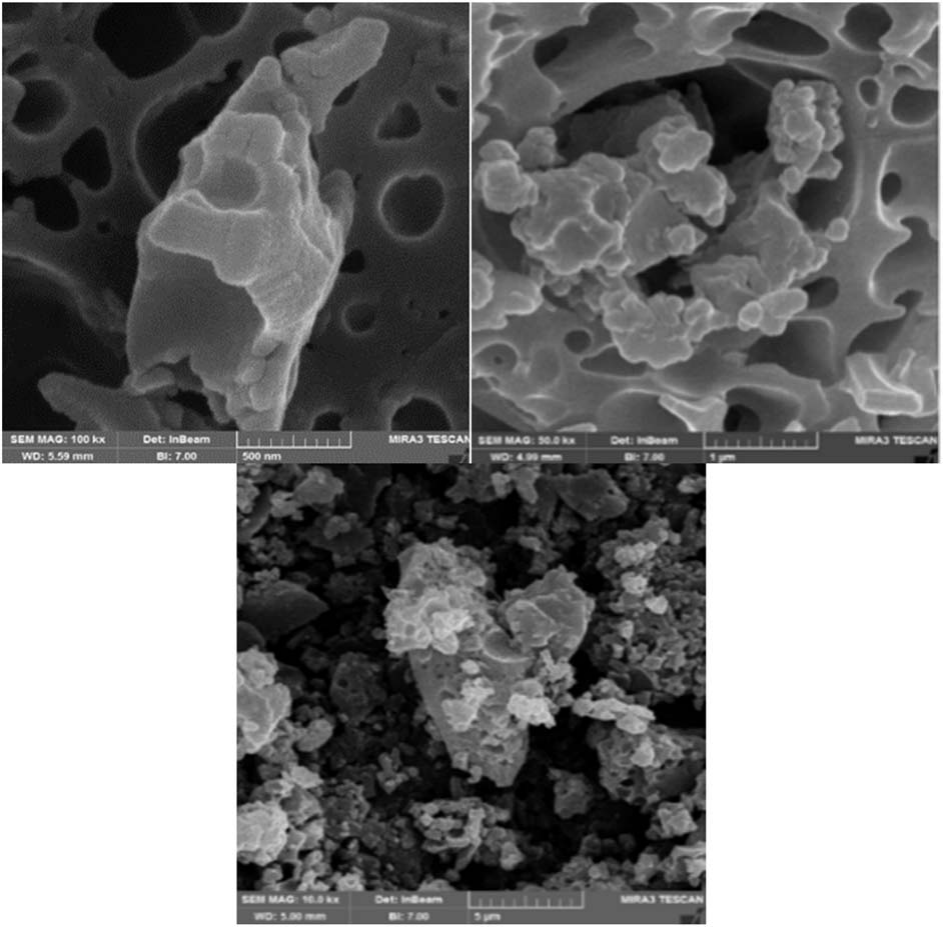
SEM images of NA-NO_2_.

The morphology and particle size of NA-NO_2_ were determined using a scanning electron microscope. SEM images showed that the synthesized compound has spherical particles with an average diameter of 48–102 nm (Fig. 7).

### EDX-map analysis

**Fig. 8:**
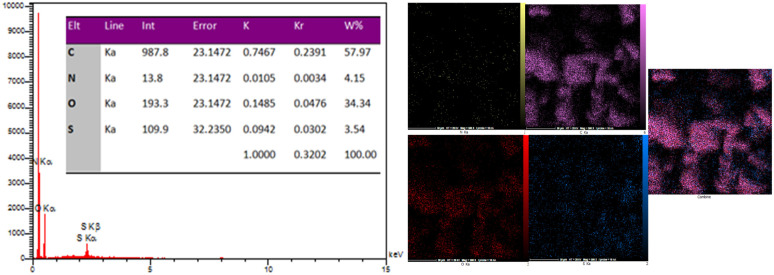
EDX and mapping analysis of NA-NO_2_.

EDX analysis of the NA-NO_2_ compound confirmed the presence of C, N, O and S elements, which are also consistent with elements obtained from elemental analysis (Fig. 8). Furthermore, the map images are in agreement for the dispersion of all the elements (C, N, O and S) with the EDX results.

### AFM analysis

**Fig. 9:**
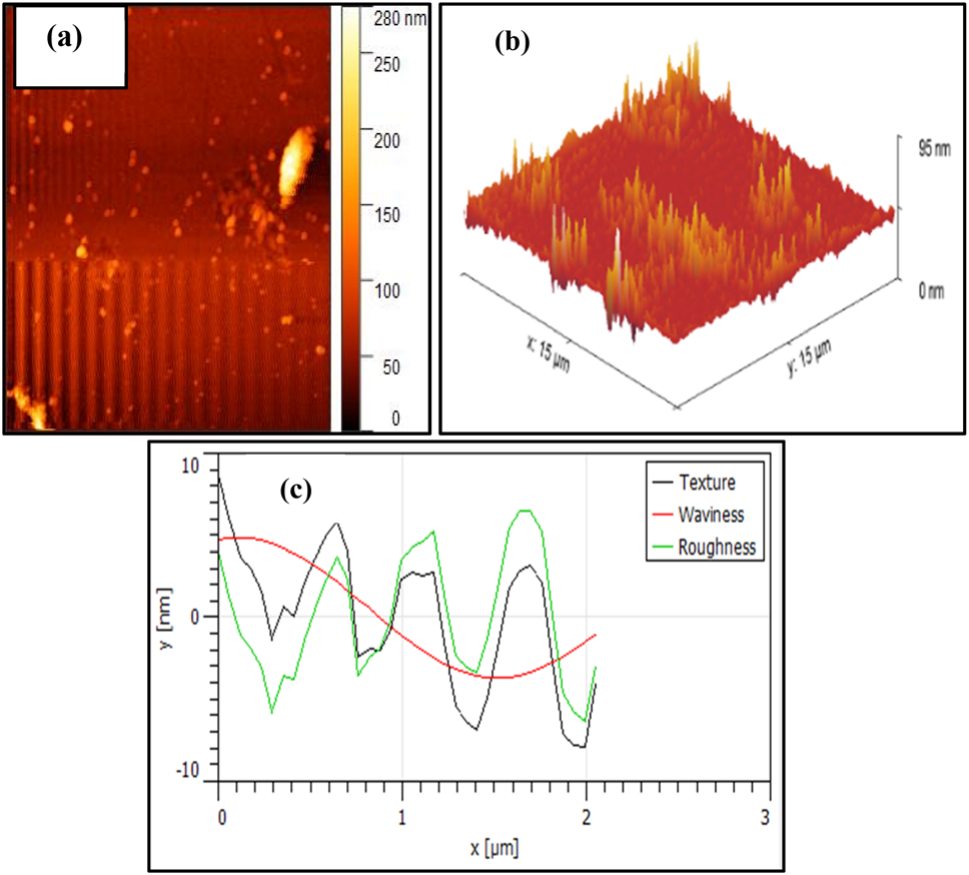
AFM images of the NA-NO_2_; (a) 2D image, (b) 3D image and (c) texture and roughness analysis.

An atomic force microscope (AFM) was utilized to investigate 2D and 3D topography, surface texture, morphology, and size of NA-NO_2_ nanoparticles. The high-grade topography of hills and valleys illustrates that it is related to the porous nature of NA-NO_2_ (Fig. 9a). In addition, the 3D image revealed an average surface RMS roughness of 56.31 nm (Fig. 9b). This average roughness is useful for calculating the average height over the entire region. The analysis of texture, waviness and average roughness of NA-NO_2_ nanoparticles in the area of 1 × 1 μm^2^ is shown in Fig. 9c.

### Band gap energy

Based on UV-vis absorption data, direct and indirect band gap energies can be obtained for energetic materials such as NA-NO_2_, PP-NO_2_ and PB-NO_2_. These calculations are defined by eqn (1):^39^

1 (*αhν*)^2^ = *A*(*hν* − *E*_g_)

where *A* and *E*_g_ are the constant values and direct/indirect band gap energy, respectively.

**Fig. 10:**
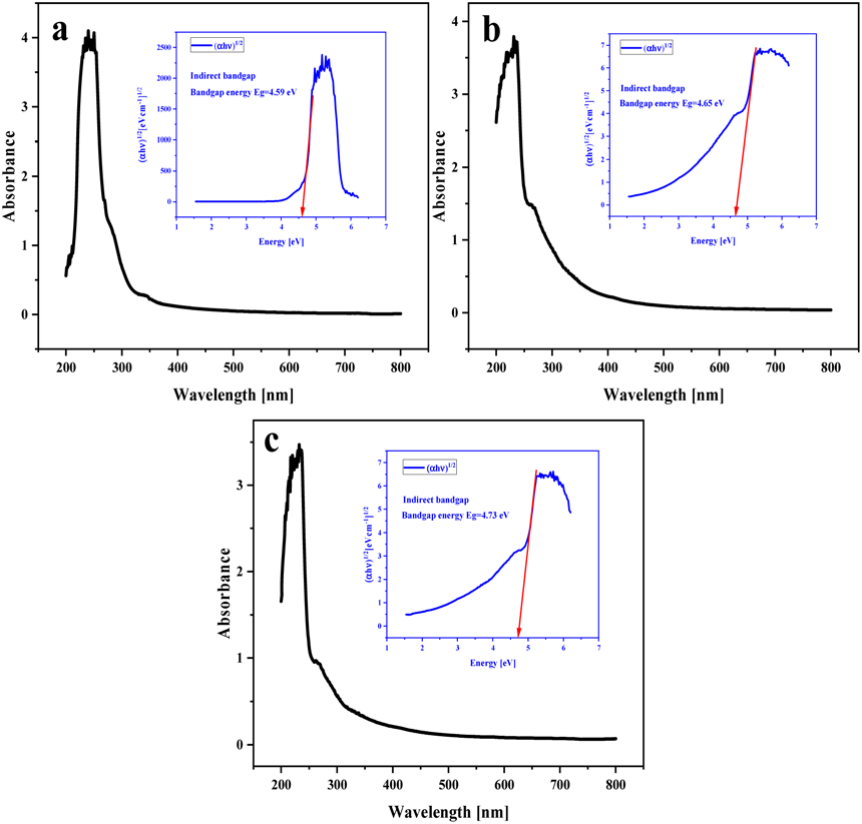
Indirect band gap energies (a) NA-NO_2_, (b) PP-NO_2_ (c) and PB-NO_2_.

Band gaps of solid bituminous nitrate hydrocarbons are shown in Fig. 10. *E*_g_ values of NA-NO_2_, PP-NO_2_ and PB-NO_2_ compounds have indirect band gaps of 4.59, 4.65 and 4.73 eV, respectively. Band gap energy calculations show that they are insulators. This exhibits that electron transfer from the valence band to the conduction band occurs more easily with a smaller band gap. NA-NO_2_ explodes faster compared to PP-NO_2_ and PB-NO_2_ due to its smaller band gap. Hence, it can be inferred that a relationship exists between explosive sensitivity and the band gap of the energetic materials based on solid bituminous hydrocarbons. This is consistent with experimental reports and software calculations. The *E*_g_ values corresponding to the direct band gap of NA-NO_2_, PP-NO_2_ and PB-NO_2_ were obtained as 4.52, 4.92 and 4.90 eV, respectively. The direct band gap *E*_g_ values of NA-NO_2_, PP-NO_2_ and PB-NO_2_ are shown in Fig. S5.†

In the gas chromatogram, the peaks corresponding to fragmented parts of the NA-NO_2_ molecule emerged at retention times of 11.977, 12.410, 24.143, 28.270 and 28.891 min (Fig. S6†). The characteristics of these fragmented molecules including molecular formula, molecular weight, proposed mass spectrum structure, base peak and peak height are shown in Table 4. The peaks of the fragmented molecules that appeared at different retention times may be due to their polarity changes. According to the results, the presence of these molecules was more probable in the synthesized sample, which was consistent with the GC chromatogram and the data obtained from the mass spectra. The mass spectra of fragmented molecules in NA-NO_2_ are shown in Fig. S7.† Proton nuclear magnetic resonance (^1^H-NMR) spectroscopy is a powerful tool in organic chemistry for identifying and analyzing molecular structures. According to the assignment of ^1^H-NMR spectrum of NA-NO_2_ the ratio of aliphatic, alkene and aromatic hydrogens was determined as 8 : 1 : 1 (Fig. S8†). The ^1^H-NMR spectrum shows that NA-NO_2_ could be considered an organic hydrocarbon.

**Table 4:** Summary of MS results for the fragmented molecules obtained from the NA-NO_2_ sample

Entry	R.T.^a^ (min)	MF^b^	MW^c^	Proposed MS^d^	Base peak	Peak height	% Of total
1	11.977	C_15_H_24_O	220	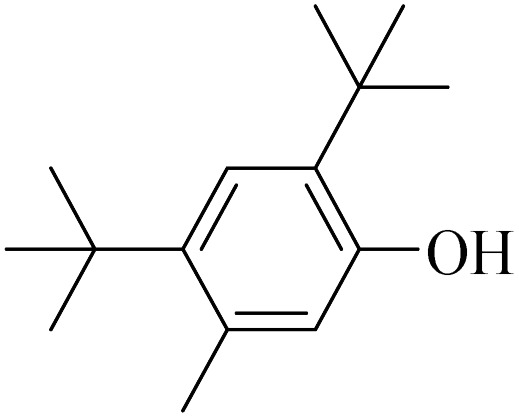	205	53 090	11.914
2	12.410	C_15_H_24_O	220	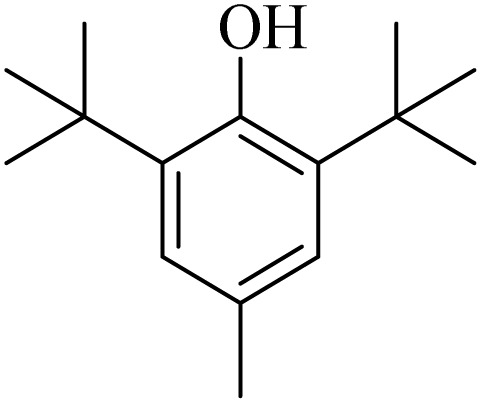	205	103 579	22.059
3	24.143	C_14_H_31_BO_6_Si_2_	362	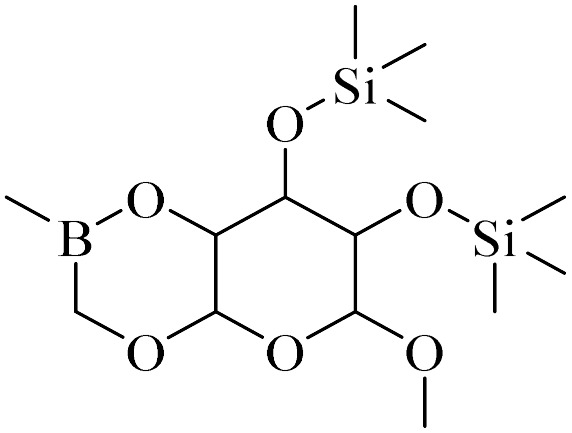	75	172 864	14.579
4	28.270	C_11_H_16_O_4_	212	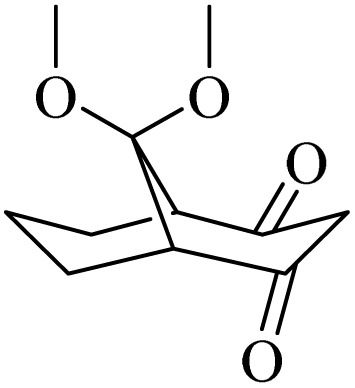	43	22 399	1.956
5	28.891	C_12_H_22_Si_2_	222	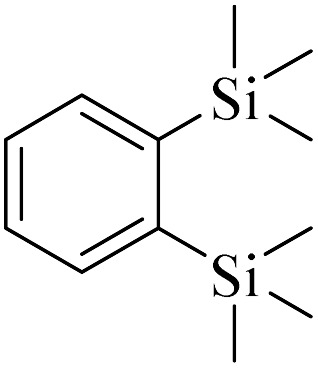	207	36 283	4.159

a Retention Time.

b Molecular Formula.

c Molecular Weight.

d Mass Spectrometry.

## Experimental section

### Materials and apparatus

Solvents and chemicals were purchased from Merck chemical company, and natural asphalt was prepared from natural bitumen mines in the west of Iran. Pitch powder with 200 mesh from Bandar Abbas Refinery and petroleum bitumen with 60–70 grade was prepared from Jey Isfahan Refinery. Fourier transform infrared spectroscopy (FTIR) was performed by the KBr pellet method by FTIR (VERTEX 70, Bruker, Germany) spectroscopy. Agilent Technologies, Cary Series UV-vis Spectrophotometer, American, was used to measure the absorption of synthesized nitro-containing samples and their substrates. Thermogravimetric analysis (TGA) was conducted from ∼30 to 800 °C using TGA (NETZSCH, Germany). The nitrogen adsorption–desorption isotherms were measured on a Micromeritics, Asap2020, USA. unit at 77 K. Scanning electron microscopy (SEM) images were examined by FE-SEM on a TESCAN MIRA III (Czechia). The heat of combustion was measured by the ASTM-D240 test method using a calorimeter bomb. The content of density was determined using the ASTM-D8176 method at room temperature. Atomic force microscopy (AFM) images were collected on an ENTEGRA AFMNT, NT-MDT, Russian, instrument, and an elemental analysis was performed to determine % C, % H, % N, and % S using COSTECH-4010, Italy, instrument. GCMS analyses were performed with an Agilent 6890 technologies series Gas Chromatograph (GC) and Agilent 5973 Mass Spectrometer (MS). The GC separations were performed with an HP-5MS column (30 m × 250 μm × 0.25 μm) and helium gas (1 mL min^−1^).

Before the GCMS analysis, a quantity (1 μL) of each sample was dissolved in dimethyl sulfoxide (1 mL) and an aliquot (1 μL) of this solution was introduced by the splitless mode. The inlet heater was fixed at 270 °C, the pressure at 7.65 psi and the total flow was 41 mL min^−1^. The oven temperature was initially kept at 50 °C for 0 min, then heated at a linear rate of 10 °C min^−1^ until 290 °C, and eventually kept at this temperature for 30 min. The MS quadrupole temperature was set at 150 °C and the MS source temperature was 230 °C. The solvent delay time was 10 min. The MS was operated in the EMV mode (absolute).

#### Physicochemical and energetic properties

The physicochemical and energetic properties of NA-NO_2_, PP-NO_2_ and PB-NO_2_ as new energetic materials, including thermal stability, heat of combustion (Δ*H*) and density were investigated. Moreover, explosive parameters such as velocity of detonation, detonation pressure, detonation temperature, heat of detonation based on liquid H_2_O and vapour H_2_O, Average molecular weight of gaseous products, moles of gaseous per gram of explosive and oxygen balance were calculated using EMDB V 1.0 (ref. 40) software (Table 5).

**Table 5:** Physicochemical and energetic properties of NA-NO_2_, PP-NO_2_ and PB-NO_2_

Compound	*T*_d_ a [°C]	VOD^b^ [m s^−1^]	*P*_cj_ c [GPa]	*T*_det_ d [K]	*Q*_det_ e [kJ mol^−1^]	*M* f [g]	*α* g	OB^h^ [Ω]	Δ*H*^i^ [kJ kg^−1^]	*D*_p_ j
NA-NO_2_	338	4830	5.1	5738	2.86	26.56	0.0297	−66.95	23 500	3.06 N_2_ + 36.78 CO + 10.13 C(s) + 2.34 H_2_
PP-NO_2_	588	4650	4.2	6278	2.59	27.175	0.0271	−71.93	23 450	3.34 N_2_ + 24.5 CO + 25.92 C(s) + 3.71 H_2_
PB-NO_2_	590	4150	2.3	5236	1.87	24.95	0.0217	−88.28	23 360	4.09 N_2_ + 33.97 CO + 15.02 C(s) + 1.26 H_2_

a Decomposition temperature by DSC (heating rate of 10 °C min^−1^).

b Velocity of detonation.^41^

c Detonation pressure.^42^

d Detonation temperature.^43^

e Heat of detonation based on Liquid H_2_O and Vapour H_2_O.^41^

f Average molecular weight of gaseous products.^41^

g Moles of gaseous per gram of explosive.^41^

h Oxygen balance.^44^

i Heat of combustion (measured by bomb calorimetry).

j The combustion reaction of the synthesized compounds (calculated EMDB V 1.0).

The heat of combustion of NA-NO_2_, PP-NO_2_ and PB-NO_2_ was measured using a calorimeter bomb according to the ASTM D240 method. The heat of combustion is defined as the heat evolved using molecular oxygen when it is converted to standard oxidation products. NA-NO_2_, PP-NO_2_ and PB-NO_2_ produced 23 500, 23 450 and 23 360 kJ kg^−1^ heat of detonation, respectively (Table 5). In this way, NA-NO_2_ with 23 500 kJ kg^−1^ released energy is superior compared to some common energetic materials such as TNT, RDX, and HMX with released energies of 14 784 kJ kg^−1^, 8795.7 kJ kg^−1^ and 9232.6 kJ kg^−1^, respectively.^14^ These results confirmed that NA-NO_2_ has a higher heat of combustion compared to TNT, RDX and HMX. The density of NA-NO_2_ was measured by the ASTM-D8176 method and it was found to be 0.5 g cm^−3^ at room temperature. But the densities of TNT, RDX and HMX increase in the order: 1.65 < 1.80 < 1.91 g cm^−3^ (Tables 5 and 6).^45^ These differences confirmed that NA-NO_2_ has a lower density compared to them, so the nitrated natural bitumen compound can be considered among the lightest energetic compounds with low density.

**Table 6:** Comparison of physicochemical and energetic properties of NA-NO_2_ with TNT, RDX and HMX^45^

Compound	*T*_d_ [°C]	VOD [m s^−1^]	*P*_cj_ [Gpa]	OB [Ω]	Δ*H* [kJ kg^−1^]	*D* a [g cm^3^]	IS^b^ [J]
NA-NO_2_	338	4830	5.1	−66.95	23 500	0.5	—
TNT	290	7303	21.3	−73.97	14 784	1.65	15 (ref. 46)
RDX	210	8795	34.9	−21.61	8795.7	1.80	7.5 (ref. 46)
HMX	280	9144	39.2	−21.61	9232.6	1.91	7 (ref. 46)

a Density.

b Impact sensitivity.

Another detonation parameter that is utilized for energetic materials is oxygen balance (OB). The progress of oxidation and combustion requires a sufficient amount of OB, the positive value of which leads to excellent detonation performance. Based on Δ*H* and OB parameters, it can be concluded that NA-NO_2_ burns faster than PP-NO_2_ and PB-NO_2_. The value of OB for TNT and two compounds RDX and HMX are −73.97 and −21.61, respectively. The differences in the above values can indicate that NA-NO_2_ performs superior to PP-NO_2_, PB-NO_2_ and TNT but poorer than RDX and HMX.^45^ These values are listed in Table 6. Comparing the detonation pressure of NA-NO_2_, PP-NO_2_ and PB-NO_2_, it shows that NA-NO_2_ is stronger than the other two compounds. However, they have a lower detonation pressure than TNT, RDX and HMX (Tables 5 and 6). The percentages of C, H, N, S and O elements in NA-NO_2_, PP-NO_2_ and PB-NO_2_ were obtained through CHNSO. Using these values, OB was calculated through the specified reference given in Table 5. Given that natural asphalt is a complex mixture primarily composed of hydrocarbons along with various functional groups, traces of metals and impurities, calculating equilibrium conditions can indeed be challenging. Therefore, the following empirical formula (C_*a*_H_*b*_N_*c*_O_*d*_)^47^ was applied to calculate the OB of NA-NO_2_, PP-NO_2_ and PB-NO_2_, ignoring impurities.
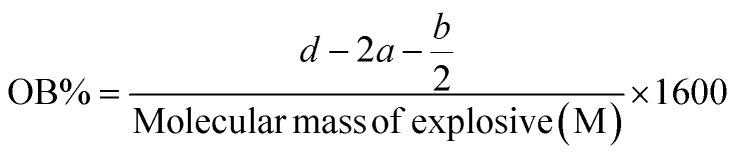
In addition, OB calculations related to NA-NO_2_, PP-NO_2_ and PB-NO_2_ are summarized in Table S1.†

Other parameters including decomposition temperature, detonation velocity and impact sensitivity of NA-NO_2_, PP-NO_2_ and PB-NO_2_ were compared with energetic materials such as TNT, RDX and HMX. The results exhibit that the high decomposition temperatures of NA-NO_2_, PP-NO_2_ and PB-NO_2_ are 338, 588 and 590 °C, respectively. More values of decomposition temperatures can indicate the thermal stability of NA-NO_2_, PP-NO_2_ and PB-NO_2_. This thermal stability can be attributed to pyrrole rings and π-conjugated structures in the synthesized energetic compounds. The impact sensitivity of NA-NO_2_, PP-NO_2_ and PB-NO_2_ was assayed and it showed that they are insensitive toward mechanical stimuli. The German Bundesanstalt für Mterialprüfung (BAM) was used to determine and calculate the impact sensitivity of NA-NO_2_, PP-NO_2_ and PB-NO2. While the impact sensitivity is generally used for determining the sensitivity of pure materials. It was found that the products are not sensitive even under heavy weights. The results showed that there was no explosion with the BAM test. While impact sensitivity of TNT, RDX and HMX decreases in the following order: 15 < 7.5 < 7 (Tables 5 and 6).

#### TG-DSC analysis

**Fig. 11:**
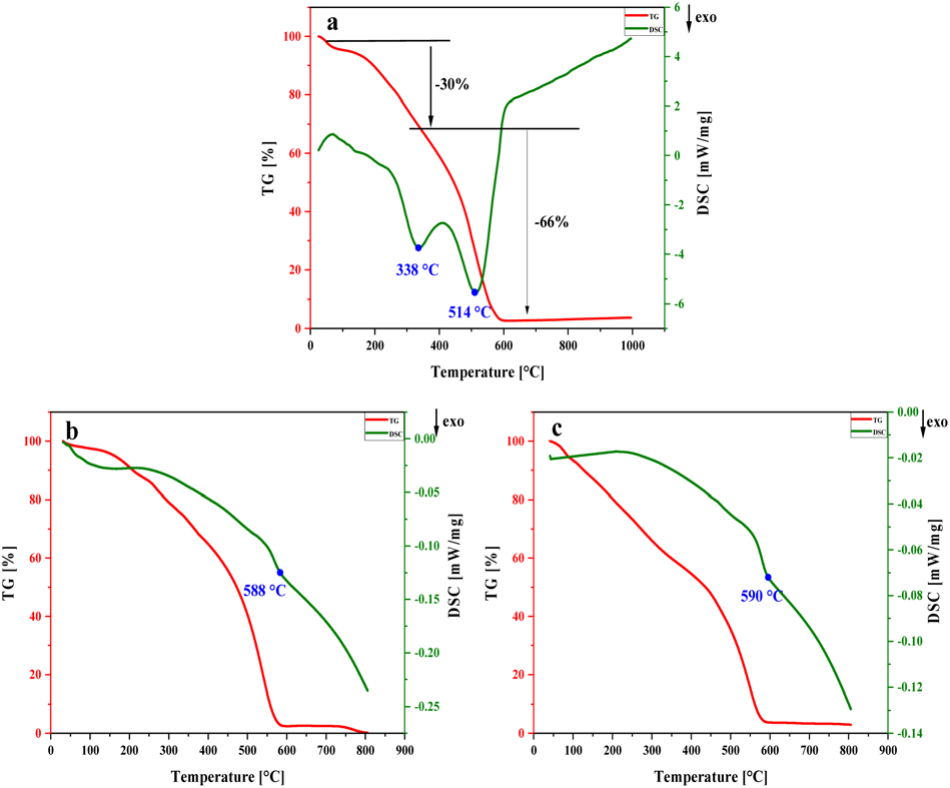
TG-DSC curves of NA-NO_2_ (a), PP-NO_2_ (b) and PB-NO_2_ (c).

The thermal behavior of TG-DSC of NA-NO_2_, PP-NO_2_ and PB-NO_2_ were applied to the thermal decomposition performance. Fig. 11 illustrates the thermal decomposition curves of NA-NO_2_ (a), PP-NO_2_ (b) and PB-NO_2_ (c). It can be seen from Fig. 11a that the thermal decomposition of NA-NO_2_ includes two stages: in the first stage at 338 °C, an exothermic peak occurs, which is attributed to the decomposition at low temperature with a 30% mass loss. This stage of decomposition is related to the process of evaporation and sublimation. In the second stage, the exothermic peak appears at the temperature of 514 °C, which is dedicated to the complete decomposition of NA-NO_2_, where 66% of the mass has been lost. Fig. 11b and c are TG-DSC curves PP-NO_2_ and PB-NO_2_, respectively. The results present that both compounds go through an exothermic stage. The TG-DSC curves of PP-NO_2_ and PB-NO_2_ show similar decomposition processes. The exothermic peak in PP-NO_2_ and PB-NO_2_ appeared at 588 and 590 °C, respectively. At temperatures lower than 588 °C in PP-NO_2_ (b), a weight loss of about 47% was observed due to the water removal, evaporation and sublimation processes. In the case of PB-NO_2_ (c), these processes occurred before 590 °C with a mass loss of 46%. In both compounds, after the exothermic peaks of PP-NO_2_ and PB-NO_2_, the mass decreased by 52% and 51%, respectively, which corresponds to their complete decomposition.

## Conclusions

To summarize, three solid bituminous hydrocarbons *i.e.* natural asphalt, petroleum pitch and petroleum bitumen were nitrated for the first time by a simple, low-cost and common HNO_3_/H_2_SO_4_ method. The nitrification reaction leads to the production of 3, 2.75 and 2.6 g and yields more than % 100 of energetic materials NA-NO_2_, PP-NO_2_ and PB-NO_2_ from 2 g of the substrate materials, respectively. Additionally, to explain the solubility behavior of NA-NO_2_, PP-NO_2_ and PB-NO_2_ compounds, diverse solvent systems were analyzed, and it was found that they were completely dissolved in aprotic solvents such as DMSO. Also, these compounds decompose at temperatures above 300 °C. The results show that these energetic materials are thermally stable and insensitive to mechanical stimuli. NA-NO_2_, PP-NO_2_ and PB-NO_2_ evolved very good heat of combustion of 23 500, 23 450 and 23 360 kJ kg^−1^, respectively. These features indicate that bitumen and its derivatives have the potential to be used as stable and insensitive energetic materials. We believe that according to the physicochemical properties and thermal behavior and the importance of the availability of cheap raw materials for the synthesis of energetic materials, the nitrated bituminous newly synthesized compounds can be expanded in a wide range of prospective applications in the field of other energetic materials.

## Data availability

The data that support the findings of this study are available in the ESI.†

## Author contributions

S. A.: validation, investigation, methodology, writing—original draft. M. S.: funding acquisition, supervision, project administration, conceptualization, resources.

## Conflicts of interest

The authors declare no competing financial interest.

## Supplementary Material

RA-014-D4RA06329E-s001

RA-014-D4RA06329E-s002
