# Supplementary material for: Synthesis of new-type, cost-effective and insensitive energetic materials via nitration of solid bituminous hydrocarbons
Source: RSC Adv. 2024 Nov 11;14(48):35971–9. doi: 10.1039/d4ra06329e (PMC11551920; doi:10.1039/d4ra06329e)
Supplement: RA-014-D4RA06329E-s002 [file RA-014-D4RA06329E-s002.pdf]

Supplementary Information (SI) for RSC Advances.
This journal is © The Royal Society of Chemistry 2024

RSC Advances

Supporting Information

Synthesis a new type, cost-effective and insensitive energetic materials by nitration of solid bituminous hydrocarbons

Sahar Abdolahi and Mohammad Soleiman-Beigi*
Department of Chemistry, Faculty of Basic Sciences, Ilam University, P.O. Box 69315516, Ilam, Iran.

E-mail: SoleimanBeigi@yahoo.com; m.soleimanbeigi@ilam.ac.ir

TABLE OF CONTENTS

Contents **Page**

Table 1 ....S3

Table S1. OB values of $NA-NO_2$, $PP-NO_2$ and $PB-NO_2$……………………………………S4
Figure S1. FT-IR spectra of NA (a), $NA-NO_2$ (with only HNO_3) (b), $NA-NO_2$ (5 mL AC_2O) (c), $NA-NO_2$ (10 mL AC_2O) (d) and $NA-NO_2$ (10 g P_2O_5) (e). S5

Figure S2. FT-IR spectra of NA (a), $NA-NO_2$ (HNO_3/H_2SO_4 with acid ratio 2:3) (b), $NA-NO_2$ (HNO_3/H_2SO_4 with acid ratio 4:5) (c). S6

Figure S3. UV-Visible absorption spectra for PP and $PP-NO_2$. S7

Figure S4. UV-Visible absorption spectra for PB and $PB-NO_2$. S8

Figure S5. Direct band gap energies $NA-NO_2$ (a), $PP-NO_2$ (b) and $PB-NO_2$ (c).. ....S9

Figure S6. GC Chromatogram for $NA-NO_2$. S1

Figure S7. Mass spectra of fragmented molecules in $NA-NO_2$. S11

Figure S8. ^{1}H-NMR spectrum of $NA-NO_2$…………………………...………………………………………...S12

REFERENCES S13

Table 1

Nitro natural asphalt synthesis with HNO_3

17.5 mL of HNO_3 was poured into a 100 mL round-bottom flask ice bath, and then 2 g of natural asphalt was slowly added. After 30 minutes, the reaction mixture was heated to 60 °C for 5 hours. Afterwards the completion of the reaction, 300 mL of distilled water was added to it. After filtering and washing, it was dried in the oven for 3 hours at a temperature of 80 °C.

Nitro natural asphalt synthesis by HNO_3/ H_2SO_4/AC_2O (5 mL), HNO_3/ H_2SO_4/AC_2O (10 mL) and HNO_3/ H_2SO_4/P_2O_5 (10 g)

First, 2 g of natural asphalt was slowly added to the HNO_3/H_2SO_4 mixture with a ratio of (17.5 mL/20 mL) to a 100 mL round-bottom flask in an ice bath. In the next step, 5 (entry 4), 10 mL AC_2O (entry 5) and 10 g of P_2O_5 (entry 6) are added to the reaction mixture for 20 minutes. After half an hour, a temperature of 60 °C is given to it for 5 hours. The reaction mixture is poured into 300 ml of distilled water. After filtering, the precipitate obtained is kept in an oven at 80 °C for 3 hours to dry. After the synthesis of the above products, the IR spectrum was applied to identify them. Their FTIR spectra indicate that groups NO_2 of three synthesized compounds have two peaks around 1350-1550 cm^{-1}. The color of nitro products obtained from the above three methods was dark brown, brown and light brown respectively. In addition, they were completely dissolved in aprotic solvents such as DMSO and decomposed at a temperature higher than 300 °C.

Nitro natural asphalt synthesis by HNO_3/H_2SO_4/$CaCl_2$

This synthesis method is summarized as follows: 2 g of natural asphalt powder was slowly added to a mixture of HNO_3/H_2SO_4 with a ratio of 17.5 mL:20 mL to a round-bottom flask in an ice water bath. Gradually, 10 g of $CaCl_2$ was poured into it. After one hour, the reaction temperature was increased to 60 °C for 5 hours. After the completion of the reaction, we added it to 300 mL of distilled water. Then it was dried at 80 °C for 3 hours after filtering and washing. Finally, a gray precipitate was obtained which was not capable of burning and its FTIR spectrum showed that it was not nitrated.

Nitro natural asphalt synthesis by $NaNO_2$/ $KHSO_4$/ H_2O_2[1]

Natural asphalt (1 g), sodium nitrite (0.8 g) and hydrogen peroxide (0.03 g) were mixed in acetonitrile for 6 h at a temperature of 60 °C in a round-bottom flask. After the completion of the reaction, the resulting mixture was added to 300 ml of distilled water. The precipitate was filtered and placed at a temperature of 80 °C (3 hours) to dry. The FTIR spectrum of the synthesized compound demonstrated that it was not nitrated.

Table S1. OB values of $NA-NO_2$, $PP-NO_2$ and $PB-NO_2$

Sample	Formula	OB [Ω]
$NA-NO_2$	$C_{46.91}H_{4.68}N_{6.11}S_{5.52}O_{36.78}$	-66.51
$PP-NO_2$	$C_{48.99}H_{2.53}N_{8.18}S_{6.33}O_{33.97}$	-69.42
$PB-NO_2$	$C_{50.42}H_{7.42}N_{6.67}S_{10.99}O_{24.50}$	-87.67

The above results confirm that the OB obtained from the EMDB V 1.0 software related to $NA-NO_2$, $PP-NO_2$ and $PB-NO_2$ is consistent with the calculations of the mentioned formula.[2]

$$OB\% = \frac{d - 2a - {}^{b}/_{2}}{Molecular\ mass\ of\ explosive\ (M)} \times 1600$$

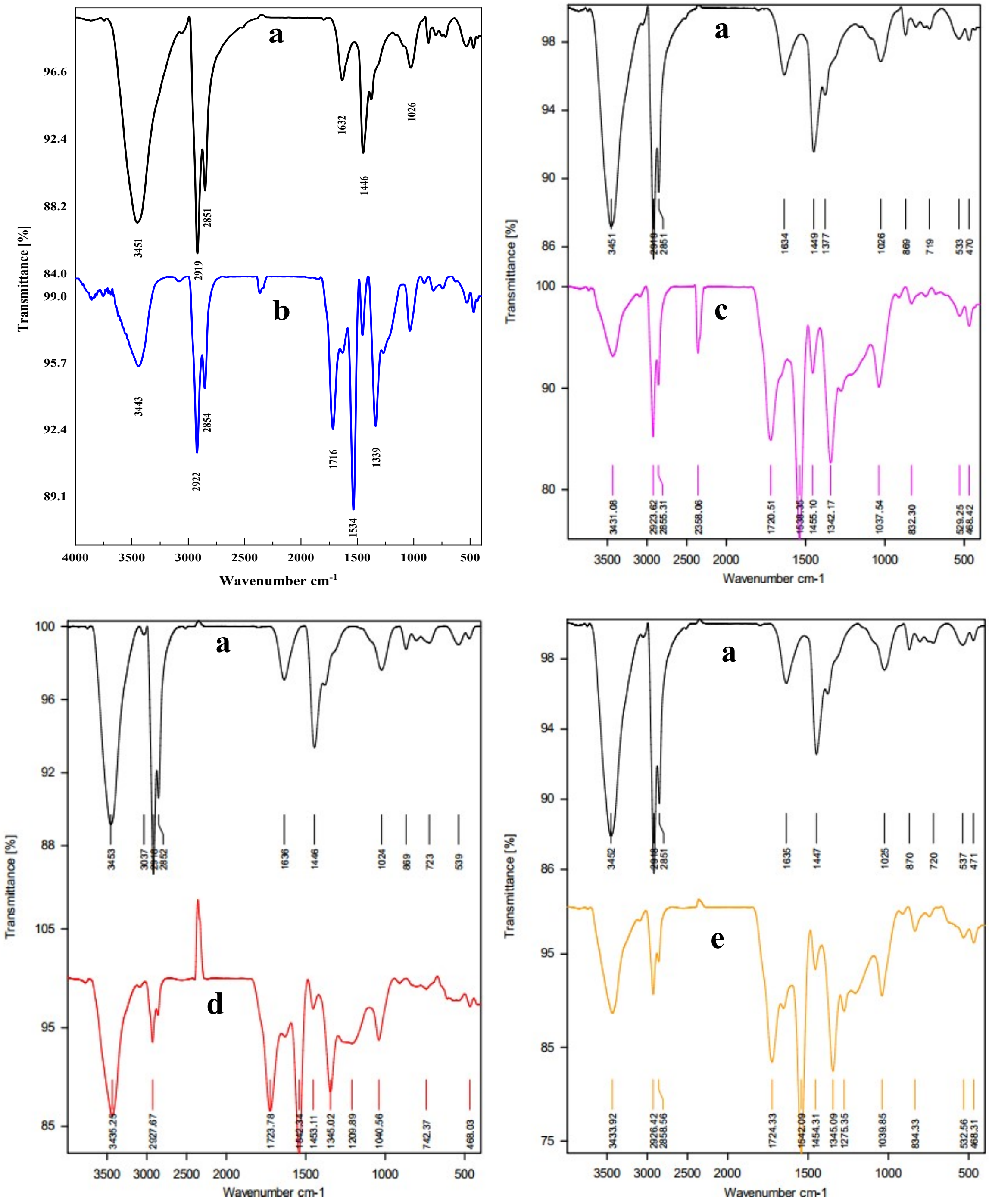

Figure S1. FT-IR spectra of NA (a), $NA-NO_2$ (with only HNO_3) (b), $NA-NO_2$ (5 mL AC_2O) (c), $NA-NO_2$ (10 mL AC_2O) (d) and $NA-NO_2$ (10 g P_2O_5) (e).

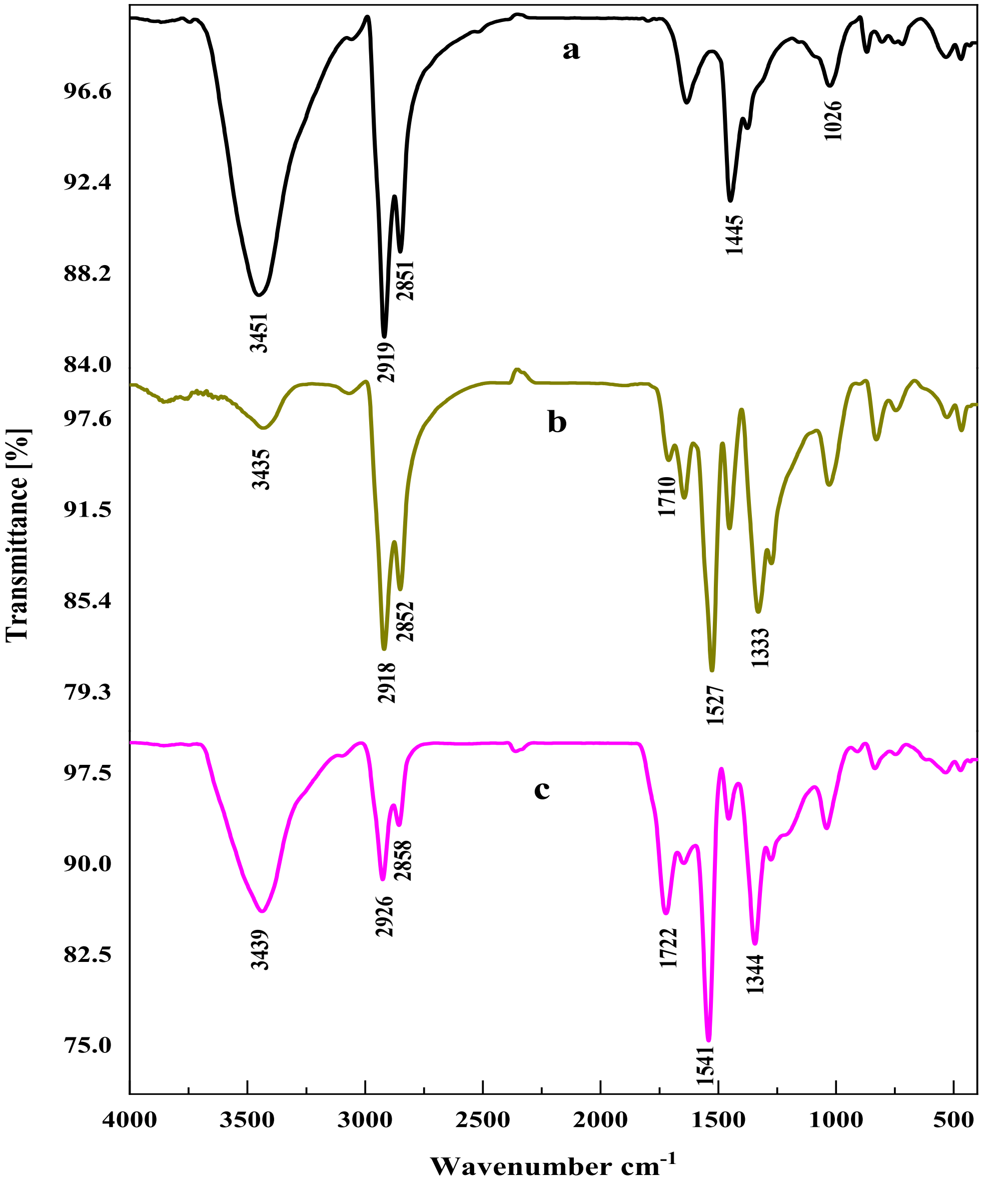

Figure S2. FT-IR spectra of NA (a), NA-NO_2 (HNO_3/H_2SO_4 with acid ratio 2:3) (b), NA-NO_2 (HNO_3/H_2SO_4 with acid ratio 4:5) (c).

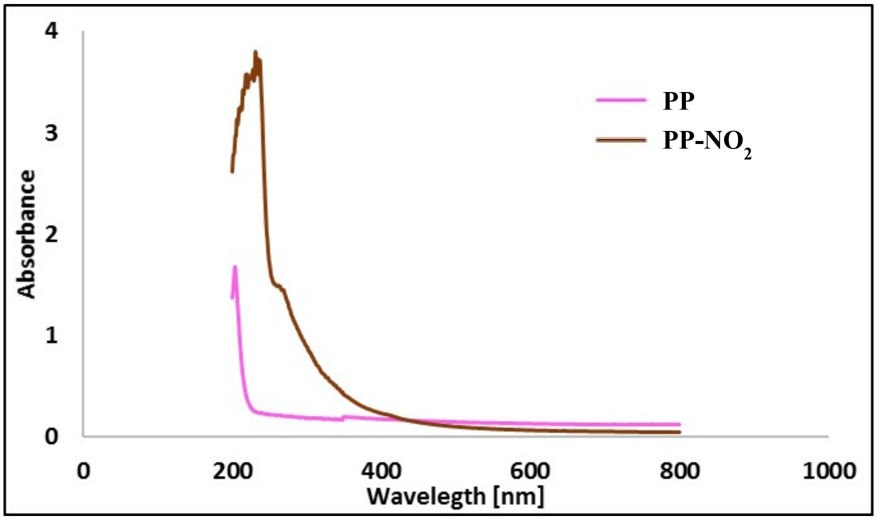

Figure S3. UV-Visible absorption spectra for PP and PP-NO_2.

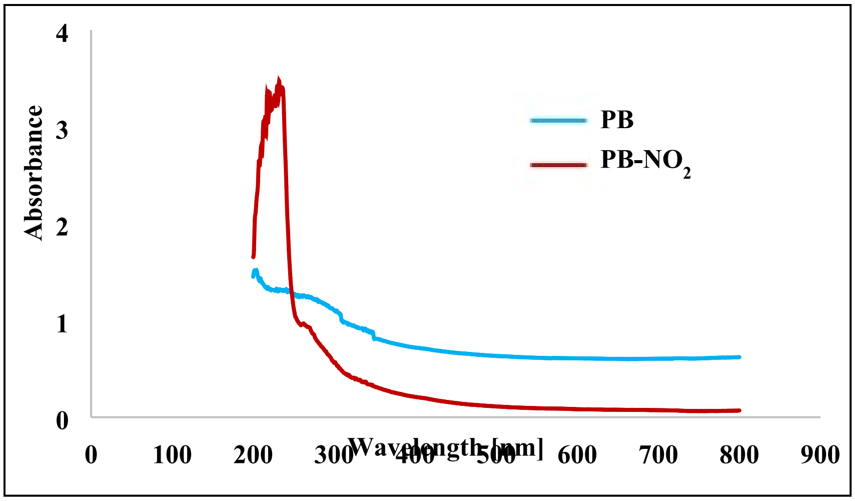

Figure S4. UV-Visible absorption spectra for PB and PB-NO_2.

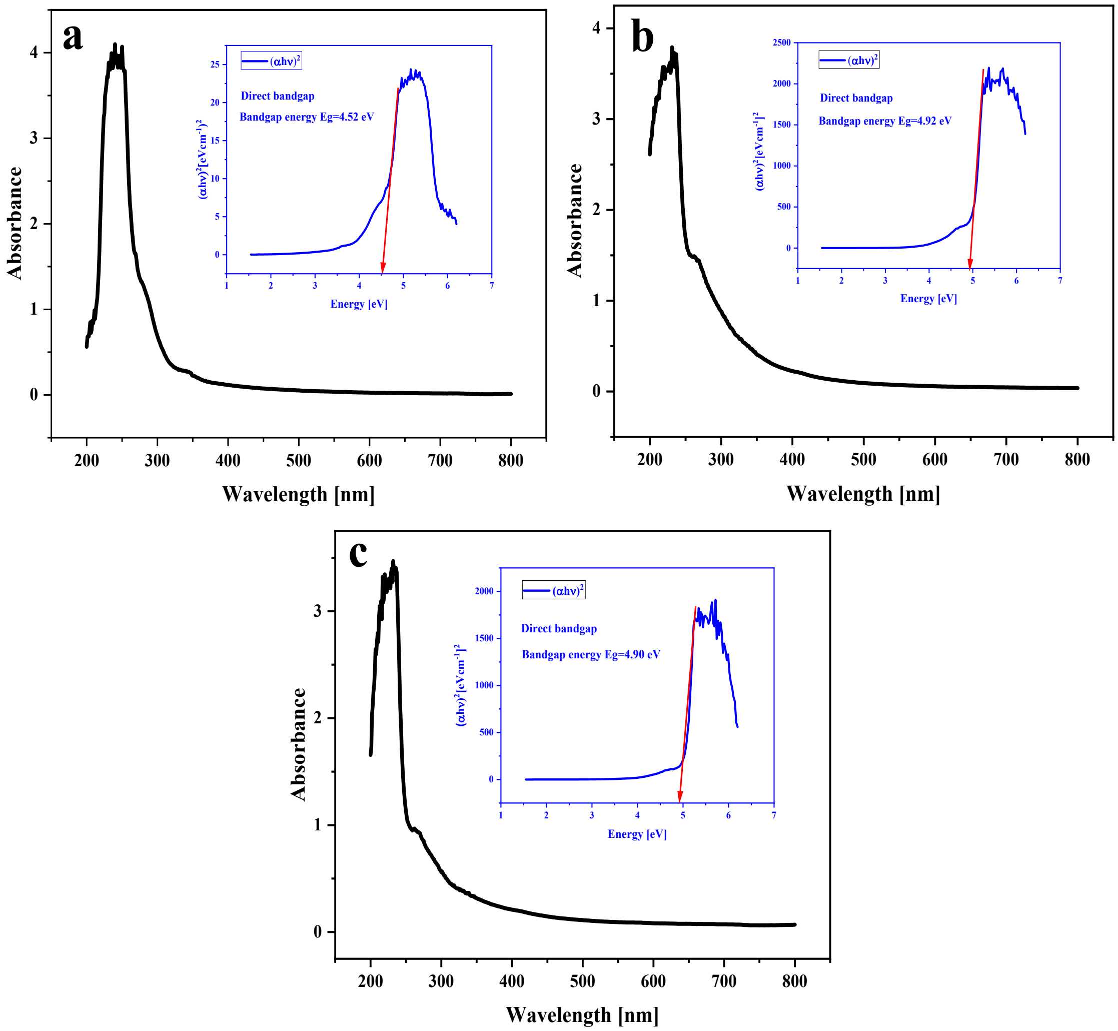

Figure S5. Direct band gap energies NA-NO_2 (a), PP-NO_2 (b) and PB-NO_2 (c).

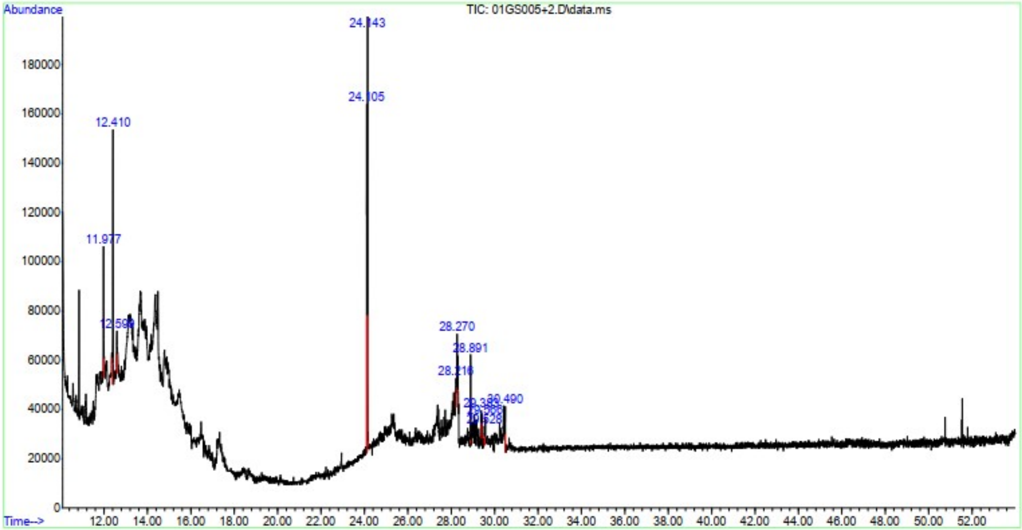

Figure S6. GC Chromatogram for NA-NO_2.

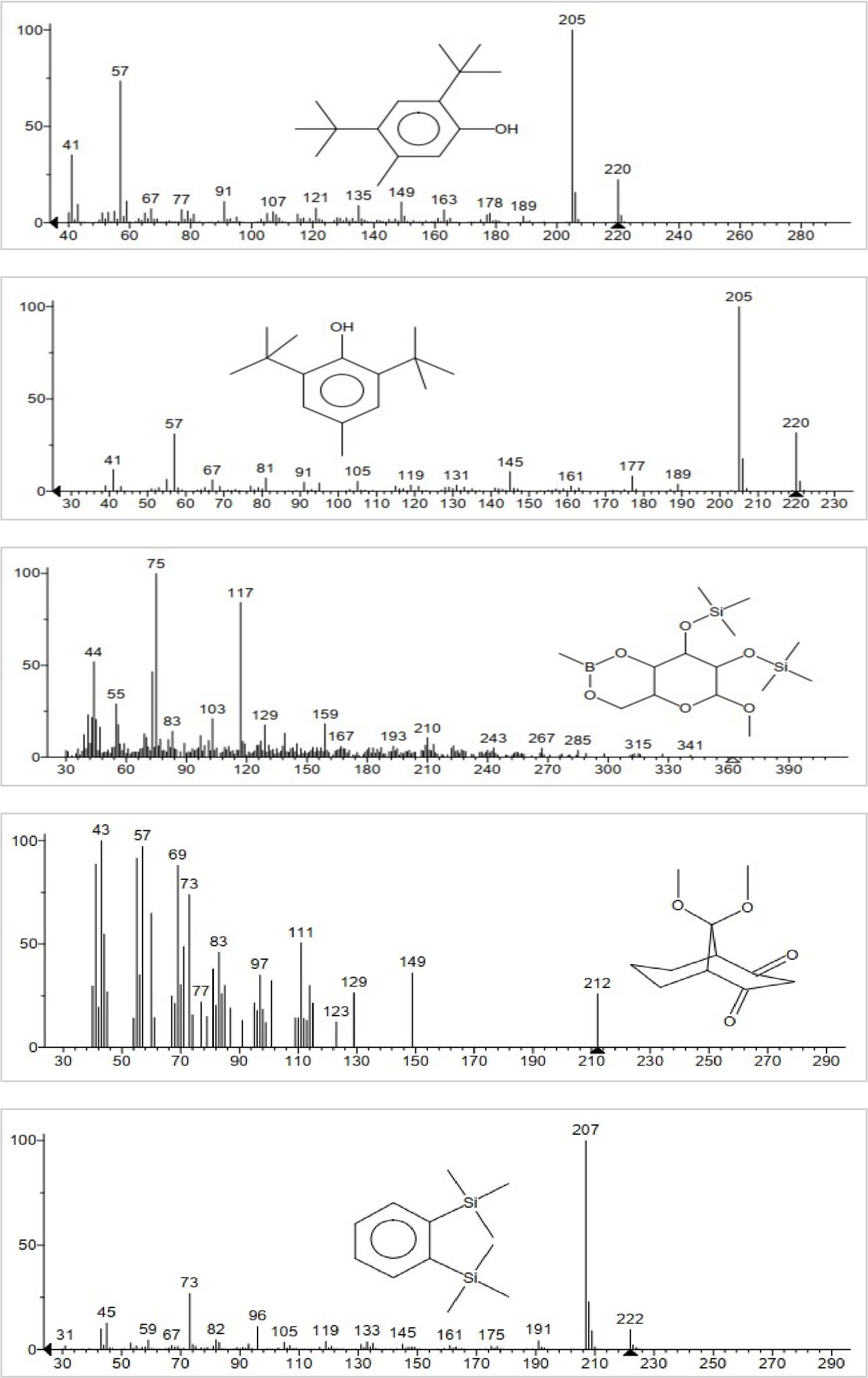

Figure S7. Mass spectra of fragmented molecules in NA-NO_2.

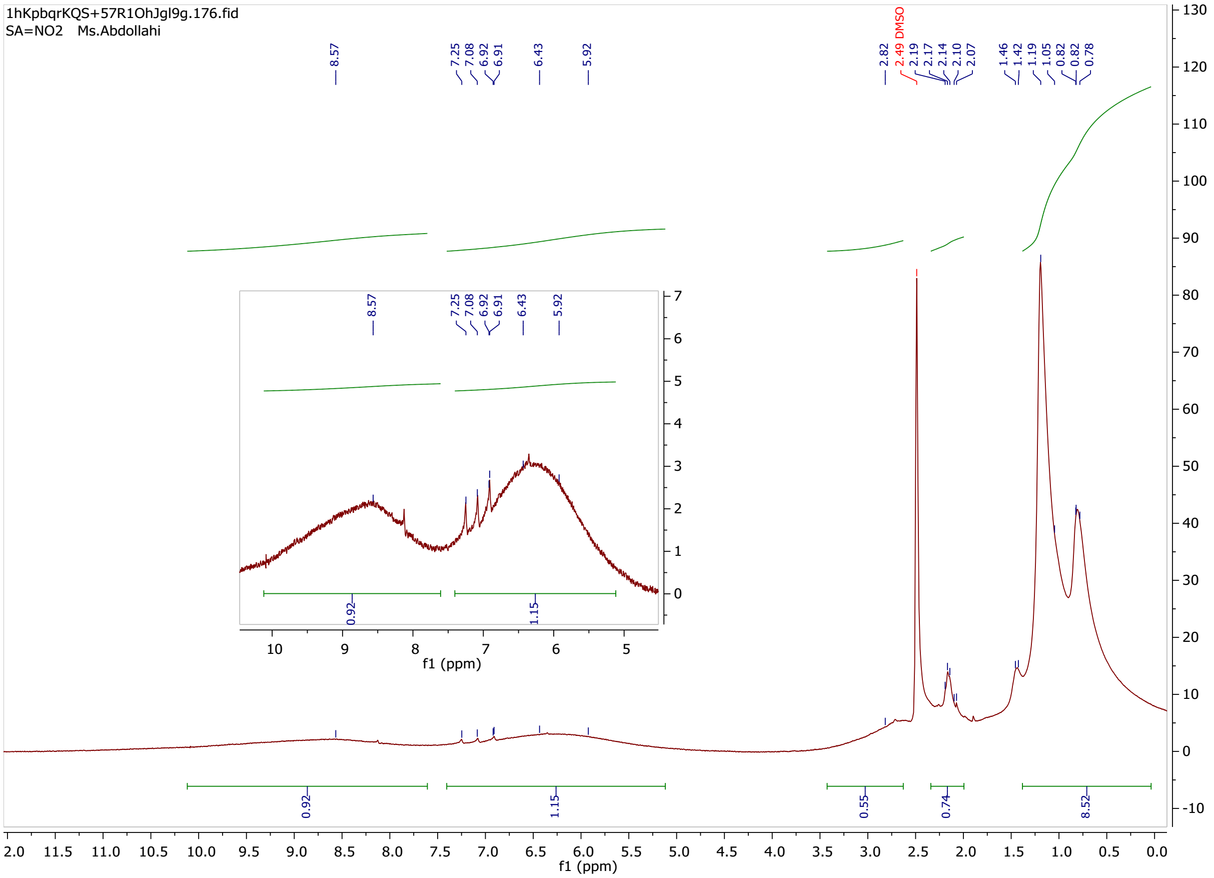

Figure S8. ^{1}H-NMR spectrum of NA-NO_2

REFERENCES

1 S. Muppidi, C. R. Kamatala, S. C. Voruvala and S. K. Mukka, Chem. Data Collect., 2019, **21**, 100222.

2 C. M. Tarver, *Propellants, Explos. Pyrotech.*, 2010, 35, 494–494.
